# Chondroid lipoma of the neck: a case report

**DOI:** 10.1186/s13104-018-3523-2

**Published:** 2018-06-28

**Authors:** Yusei Katsuyama, Toshiharu Shirai, Ryu Terauchi, Shinji Tsuchida, Naoki Mizoshiri, Yuki Mori, Toshikazu Kubo

**Affiliations:** Department of Orthopedics, Graduate School of Medical Science, Kyoto Prefectural University of Medicine, 465 Kajii-cho, Kamigyo-ku, Kyoto, 602-8566 Japan

**Keywords:** Chondroid lipoma, Sarcoma, FDG PET

## Abstract

**Background:**

Chondroid lipoma, first described in 1993 by Meis and Enzinger, is a very rare lipomatous tumor. Because it is a benign tumor, it does not require radiotherapy, chemotherapy, or extensive resection. However, histologically, it is often confused with a sarcoma. It is crucial to differentiate chondroid lipoma from sarcoma to avoid choosing an inappropriate treatment strategy. Although MRI, radiography, and ultrasound have been used to evaluate chondroid lipomas, imaging cannot accurately differentiate chondroid lipoma from sarcoma.

**Case presentation:**

A 39-year-old man presented to a local clinic with a 1-month history of a painless mass in his left neck. Results of a needle biopsy suggested an atypical lipomatous tumor, and the patient was referred to our hospital. Physical examination revealed a hard and mobile mass in the left neck. Plain X-ray radiographs showed an absence of calcification in the soft tissue mass. MRI revealed a well-defined and lobulated mass, and on T1-weighted images, the lesion showed heterogeneity, with higher signal intensity than that of muscle. On T2-weighted images, the septum had low-signal intensity. On T2-weighted fat-suppressed images, the signal of the mass was completely suppressed. The SUV_max_ of the mass on FDG PET was 1.84. An additional needle biopsy was performed, and on the basis of the results, we arrived at a diagnosis of well-differentiated liposarcoma. The mass was resected marginally. Macroscopically, the mass was encapsulated and markedly harder than well-differentiated liposarcoma. Histologically, the tumor was composed of myxoid and cartilaginous matrix, and mature fat cells and lipoblast-like cells were present. The final diagnosis was chondroid lipoma, and no recurrence was observed 1 year after surgery.

**Conclusions:**

Chondroid lipoma is an extremely rare benign soft tissue tumor that is often confused with sarcoma. It is very important to differentiate chondroid lipoma from sarcoma when the SUV_max_ value of the mass is low, even when biopsy results suggest that it is a sarcoma.

## Background

Adipocytic tumor is one of the most common soft tissue tumors. Chondroid lipoma, first described in 1993 by Meis and Enzinger [1], is a very rare adipocytic tumor. Histologically, it is often confused with sarcoma, especially liposarcoma and chondrosarcoma [2, 3]. However, it is a benign tumor, and therefore, it does not require radiotherapy, chemotherapy, or extensive resection [1, 4]. Although some reports have described the evaluation of chondroid lipoma using MRI, radiography, and ultrasound [5, 6], imaging cannot definitively differentiate between chondroid lipoma and malignant sarcoma [7, 8]. Moreover, to date, there are no reports of evaluation using FDG PET. In this report, we present the FDG PET features of a case of chondroid lipoma.

## Case presentation

**Fig. 1:**
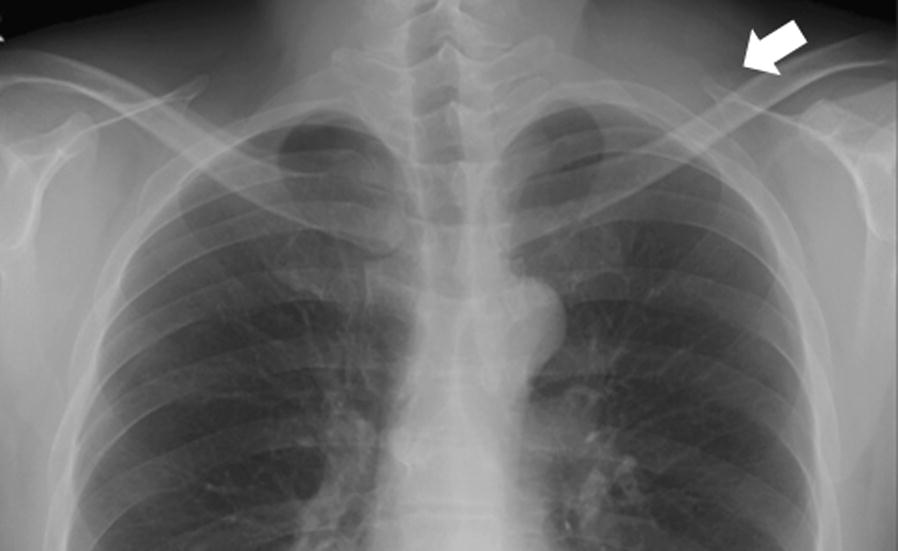
The radiographic finding show a soft tissue mass on the left neck. There is no calcification in the mass

**Fig. 2:**
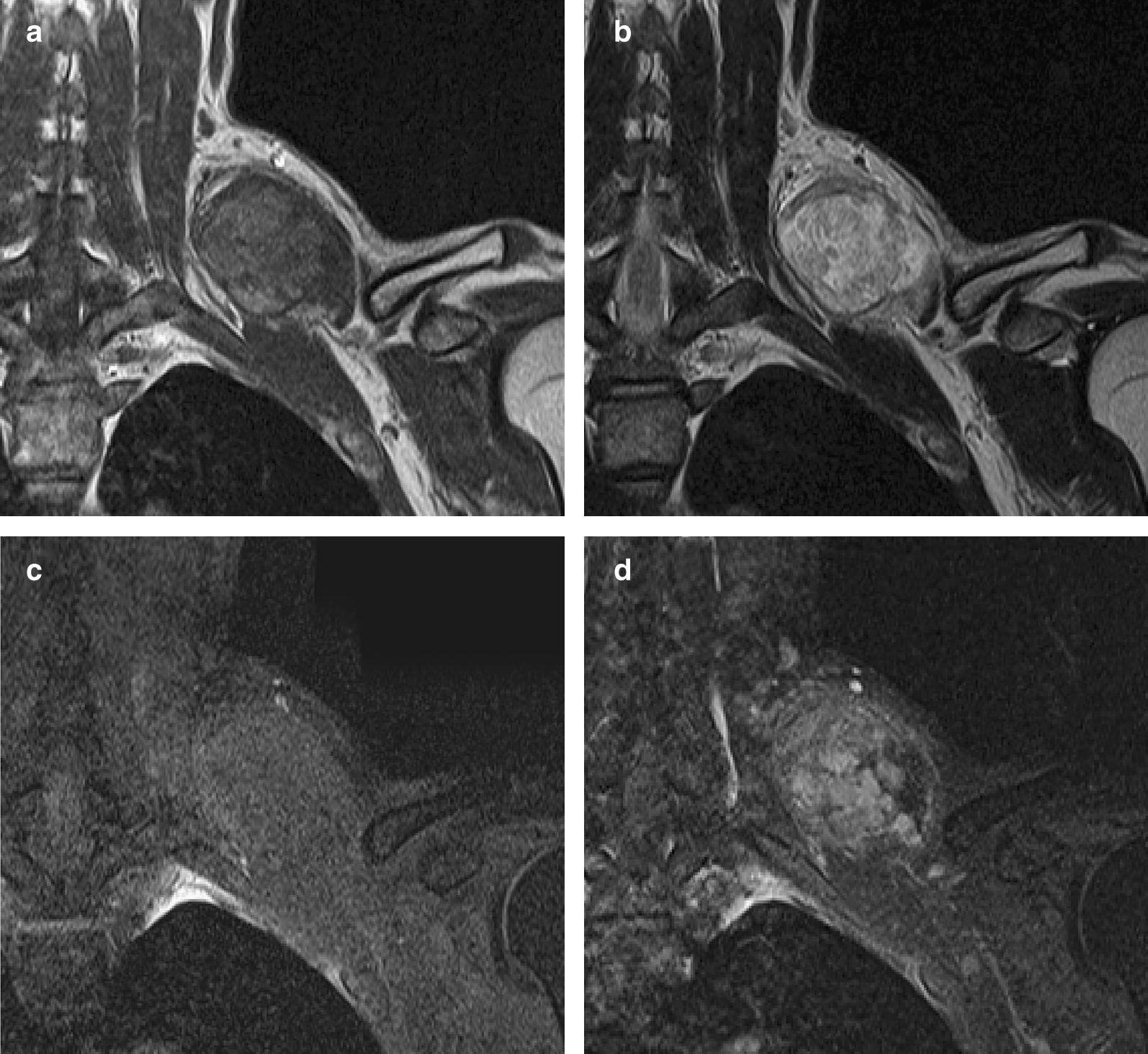
MR imaging shows a well-defined and lobulated mass on the left neck. **a** On T1-weighted images, the mass has heterogeneity with high-signal intensity than muscle. **b** On T2-weighted images, the septum has low-signal intensity. **c** On T2-weighted fat-suppressed images, the signal of the mass is suppressed totally. **d** On T1-weighted images following gadolinium administration, the mass is enhanced

**Fig. 3:**
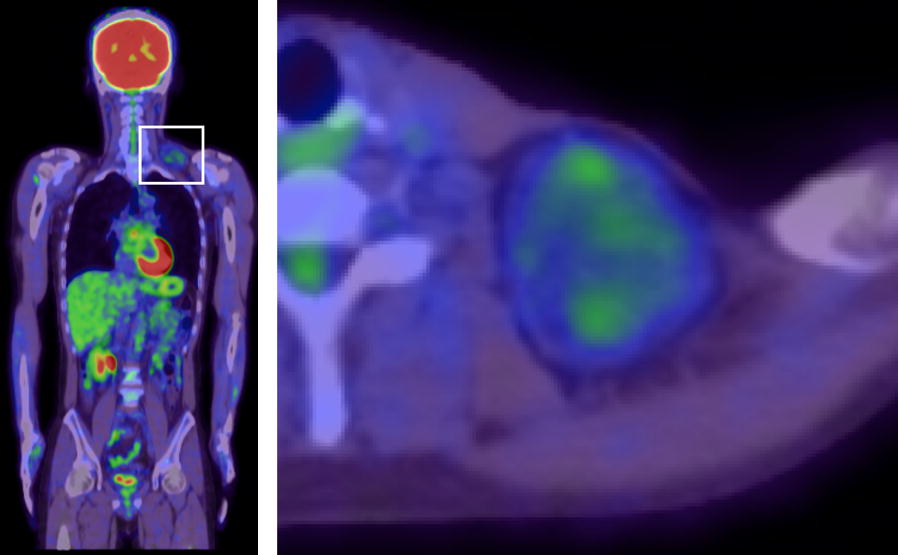
FDG PET shows that the SUV_max_ value of the mass is 1.84 and there is no abnormal uptake except for the mass

**Fig. 4:**
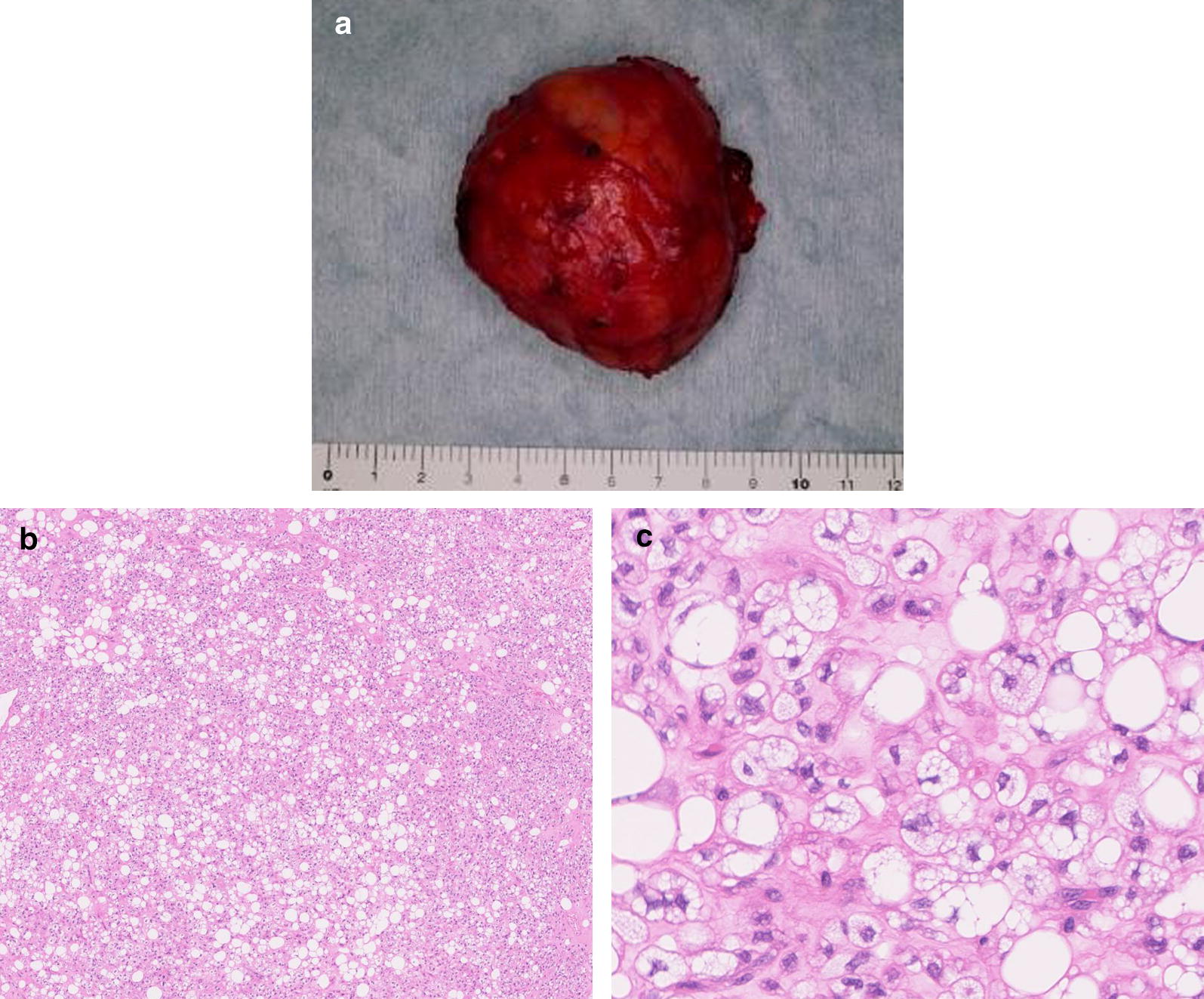
The mass after marginal resection. **a** Macroscopically, the mass is encapsulated and much harder than well differentiated liposarcoma. **b**, **c** Histologically, the tumor is composed of myxoid and cartilaginous matrix, and there are mature fat cell and lipoblast like cell

A healthy 39-year-old Japanese man presented to a local clinic with a 1-month history of a painless mass in his left neck. A needle biopsy was performed, and the results indicated the possibility of an atypical lipomatous tumor. Subsequently, he was referred to our hospital. Physical examination revealed a hard and mobile mass in the left neck, measuring approximately 10 × 10 cm. Plain X-ray radiographs showed a soft tissue mass with no calcification in the left neck (Fig. 1). MR images showed a well-defined and lobulated mass (Fig. 2a–d). On T1-weighted images, the mass had heterogeneity, with a higher signal intensity than that of muscle (Fig. 2a). On T2-weighted images, the septum had low-signal intensity (Fig. 2b). On T2-weighted fat-suppressed images, the signal of the mass was completely suppressed (Fig. 2c). On gadolinium-enhanced T1-weighted images, the signal from the mass was enhanced (Fig. 2d). The SUV_max_ value of the mass on FDG PET was 1.84, with no abnormal uptake except in the mass (Fig. 3). An additional needle biopsy was performed in our hospital, and evaluation of the results resulted in a diagnosis of well-differentiated liposarcoma. The mass was resected marginally because it was considered a low-grade tumor. Macroscopically, the mass was encapsulated and markedly harder than a well-differentiated liposarcoma (Fig. 4a). The cut surface of the mass was yellowish and lobulated. Histologically, the tumor was composed of myxoid and cartilaginous matrix, and mature fat cells and lipoblast-like cells were present (Fig. 4b, c). Immunohistochemical analysis showed that the tumor cells were negative for CDK4, MDM2, MIB1, and Sox9. On the basis of these findings, we arrived at a final diagnosis of chondroid lipoma. There was no recurrence at 1 year after surgery.

## Discussion and conclusions

Adipocytic tumor, which is one of the major categories in the WHO classification of soft tissue tumors of the neck, has many histologic subtypes [9]. Chondroid lipoma, an extremely rare benign adipocytic tumor, predominantly occurs in the proximal extremities and limb girdles [4], with a few reported in the head and neck [10]. Most patients present with a painless mass.

In previous studies of chondroid lipoma, the radiographs revealed a soft mass with calcification. The MR images showed a well-defined and lobulated mass. On T1-weighted images, the mass had heterogeneity with a high signal, which is typical of adipose tissue. On fat-suppressed sequence, the signal was completely suppressed. On T2-weighted images, the septum had low-signal intensity [5, 6]. All these findings are consistent with our findings in the present case.

Histologically, a chondroid lipoma is a well-circumscribed and lobulated tumor consisting of mature fat cells and lipoblast-like cells surrounded by a myxoid-chondroid matrix [11, 12]. It is histologically similar to liposarcoma, chondrosarcoma, myxoid liposarcoma, and osteosarcoma [2, 3].

Treatment of malignant tumors entails radiotherapy, chemotherapy, and extensive resection; however, benign tumors require only marginal resection [1, 4]. Therefore, it is very important to differentiate benign tumors from malignant tumors, including the differentiation of benign lipomatous tumors such as chondroid lipoma from malignant tumors, in order to avoid unnecessary treatment and the associated adverse effects.

Several reports have demonstrated the usefulness of diffusion-weighted MRI in the evaluation of soft tissue tumors. Razek et al. [13] showed that the apparent diffusion coefficient value of benign soft tissue tumors was significantly higher than that of malignant tumors. In contrast, another study showed that there was no significant difference in ADC values between benign and malignant tumors [14]. Surov et al. [15] reported that diffusion is determined by the balance between matrix and cellularity, but not the dignity of the lesion. Evaluation with diffusion-weighted MRI may be useful for differentiating chondroid lipoma from sarcoma.

To date, there have been no reports on the evaluation of chondroid liposarcoma by FDG PET. In the present case, FDG PET was performed to evaluate the malignancy of the tumor, and the SUV_max_ value of the mass was 1.84. Liposarcoma patients with SUV_max_ > 3.6 have a significantly worse prognosis [16]. Although not all lesions with low SUV_max_ value are benign, a low SUV_max_ value generally indicates a benign or low-grade tumor.

In addition, our findings suggest that even when a mass is diagnosed as sarcoma according to biopsy results, it is important to include chondroid lipoma in the differential diagnosis when the mass is very hard, well-defined, and lobulated, and when the SUV_max_ value of the mass is low.

In conclusion, we report a very rare case of chondroid lipoma of the neck presenting as a hard mass and diagnosed on the basis of FDG PET findings. In order to avoid overtreatment due to incorrect diagnosis, it is very important to differentiate chondroid lipoma from sarcoma when the SUV_max_ value of the mass is low, even when the mass is suspected to be a sarcoma on the basis of biopsy results.

## Abbreviations

CT: computed tomography
MRI: magnetic resonance image
FDG PET: fluorodeoxyglucose-positron emission tomography
SUV: standardized uptake value
